# Bioinspired membrane-based systems for a physical approach of cell organization and dynamics: usefulness and limitations

**DOI:** 10.1098/rsfs.2015.0038

**Published:** 2015-08-06

**Authors:** Thibaut J. Lagny, Patricia Bassereau

**Affiliations:** 1 Institut Curie, PSL Research University, Laboratory PhysicoChimie Curie, 75248 Paris, Cedex 05, France; 2 CNRS, UMR168, 75248 Paris, Cedex 05, France; 3 Université Pierre et Marie Curie, 75252 Paris, Cedex 05, France

**Keywords:** membrane, synthetic biology, reconstitution

## Abstract

Being at the periphery of each cell compartment and enclosing the entire cell while interacting with a large part of cell components, cell membranes participate in most of the cell's vital functions. Biologists have worked for a long time on deciphering how membranes are organized, how they contribute to trafficking, motility, cytokinesis, cell–cell communication, information transport, etc., using top-down approaches and always more advanced techniques. In contrast, physicists have developed bottom-up approaches and minimal model membrane systems of growing complexity in order to build up general models that explain how cell membranes work and how they interact with proteins, e.g. the cytoskeleton. We review the different model membrane systems that are currently available, and how they can help deciphering cell functioning, but also list their limitations. Model membrane systems are also used in synthetic biology and can have potential applications beyond basic research. We discuss the possible synergy between the development of complex *in vitro* membrane systems in a biological context and for technological applications. Questions that could also be discussed are: what can we still do with synthetic systems, where do we stop building up and which are the alternative solutions?

## 1. Introduction

Bioinspiration is a rich and broad topic that can be approached through many aspects. It can refer to copying nature for the synthesis of new materials (silk, organic or mineral biocomposite [1]), to use and transform nature (in general, bacteria) for producing drugs [2], fuel [3] or new chemical products [4]; these represent the usually recognized objectives of what is commonly called synthetic biology. Bioinspiration can also be a guiding line for understanding how biological systems work. Indeed, bioinspired systems can be designed in order to decipher the mechanisms behind the multiple functions that support cellular life [5,6]. This short review/opinion paper considers this class of systems only, with a particular focus on cell functions implying cell membranes such as cell trafficking, cell motility, cytokinesis, cell–cell communication, information transport in cells and between cells.

Cell biologists traditionally use top-down approaches that ‘deconstruct’ cells in a controlled way. An analogy could be made with understanding how a machine works by observing the effect of removal or transformation of some of its parts, or by affecting its energy supply. Biologists have a plethora of elaborated techniques to silence the expression of specific proteins, or to mutate them and observe the consequences on a cell's function. They can also affect the source of energy by tuning the level of adenosine triphosphate (ATP) or guanosine triphosphate (GTP) in cells. Ever more drugs are designed to target the function of cellular proteins, or to change the composition of the cellular membranes. In addition, cell observation has rapidly progressed over the years with major advances in imaging, first with the use of GFP (and its derivatives) for labelling and live imaging (Nobel Prize in Chemistry, 2008) and more recently with the advent of various high-resolution microscopy techniques (Nobel Prize in Chemistry, 2014). Developments in image analysis and also in mass spectrometry (Nobel Prize in Chemistry, 2002) together with the blooming of omics approaches allow now for a more quantitative biology. With all these approaches, biologists have provided an ever-growing understanding of membrane organization and compartmentalization, of their dynamics upon trafficking and of their role in adhesion, motility, division and signalling. However, even if the perturbations are generally focused on one type of component involved in a particular cell function, the cell response is global, and other cell functions might also be affected. It is thus difficult to isolate one mechanism at a time.

**Figure 1.**
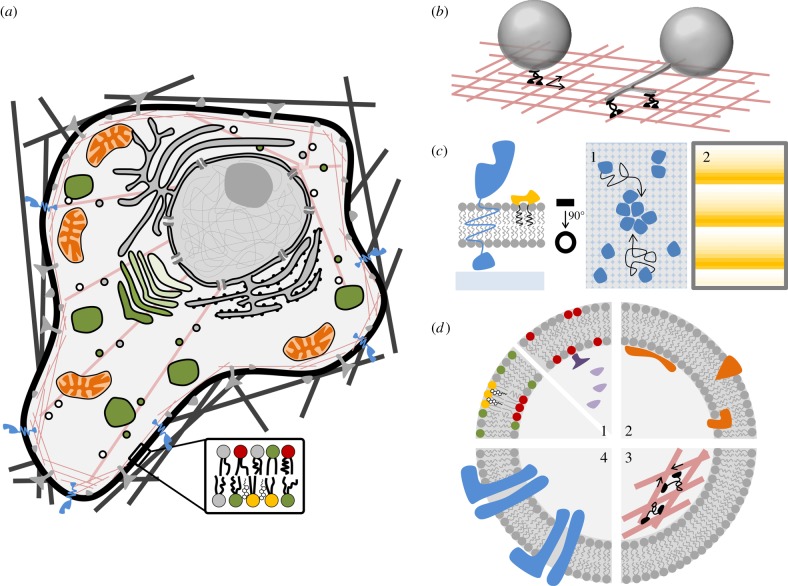
Model membrane-based systems for cell biology. (*a*) Schematic of a eukaryotic cell, highlighting the complexity that is faced when studying biological problems in living cells. (*b*). *In vitro* approaches to study vesicular transport and membrane deformation by motor proteins. The left side of the cartoon illustrates the movement of a GUV being transported by motor proteins on preassembled cytoskeletal fibres, whereas the right depicts the situation of membrane tube extrusion from GUVs by motor proteins. (*c*) Supported lipid bilayers allow for the study of membrane protein dynamics in controlled geometries and with high spatial resolution. (1) Highlights the diffusion and clustering behaviour of transmembrane receptors; (2) shows the autonomous formation of protein gradients under two-dimensional confinement. (*d*) Liposomes as biomimetic workhorses. (1) Liposomes can be formed *in vitro* with controlled lipid compositions and in discrete size ranges and allow the incorporation of purified proteins to study their lipid-binding capacity and specificity (right). They can as well be obtained from cells, allowing the study of membrane properties, e.g. phase separation, in membranes that represent the endogenous complexity of membranes independent of cytoskeletal mechanisms (left). (2) Proteins can be screened and investigated regarding their ability to induce or affinity towards membrane curvature. (3) Cytoskeletal features can be recapitulated in minimal systems, e.g. an acto-myosin network at the membrane. (4) Advanced methods allow the incorporation of transmembrane protein complexes, e.g. ion channels, or transporters.

An opposite approach consists of an engineering-type of methodology [5,7]. This bottom-up strategy has been developed by different biophysics groups in the attempt to decipher cellular mechanisms. Based on biological observations, a minimal number of biological components are identified as key for the respective cell function to analyse. A biomimetic system is then built up, which can be viewed as an isolated module of the complex cellular machinery. We review in the following the variety of membrane-based bioinspired systems that are currently used. Our intention is not to be fully exhaustive, but to provide some examples and a basis for discussion. Following this bottom-up approach, a cell can thus be decomposed into many different simplified modules (figure 1). Physicists can then use quantitative tools to characterize the interactions between these components and membranes, their organization, the mechanics of the composite protein–membrane system and its dynamics under well-defined conditions. They can also derive theoretical models and compare their predictions with the experimental results obtained on the biomimetic modules, leading eventually to a rigorous evidence for the mechanism, at least with this reduced number of elements. Naturally, these modules grow in complexity over the years, and we discuss in this opinion/review what can practically limit the bottom-up engineering strategy, but also some potential side-benefits of the development of these biomimetic model systems for applications to synthetic biology.

## 2. Model membrane-based systems for cell biology

Many model membrane systems are available for mimicking *in vitro* membrane-involving biological processes (for reviews, see [8,9]). The distinct geometries of the model systems are correlated to the experimental techniques that are used for the characterization of the biological module [10].

### 2.1. Review of the main existing systems

*Liposomes*, also called *vesicles*, are very common systems that consist of a bilayer delimiting an internal aqueous compartment from the outside. They usually have a spherical shape, but can adopt more complex shapes under certain circumstances. Depending on the preparation method, their diameter can vary over three decades:

(1) Small unilamellar vesicles (SUVs) between 30 and 100 nm. They are obtained by sonication of a hydrated preparation of lipids [11].
(2) Large unilamellar vesicles (LUVs) between about 100 nm and 1 μm. They are prepared either by extrusion through a filter of calibrated pore size [12] or by reverse-phase evaporation [13].
  Polydisperse LUVs and SUVs can be individually grafted onto a solid substrate for optical detection of the effect of membrane curvature on protein binding [14] or function [15] (single liposome curvature (SLiC) assay).
(3) Giant unilamellar vesicles (GUVs) with diameters ranging from a few micrometres to 100 μm (see [16] for a review on their potential uses). This is the best-suited liposome system for optical microscopy measurements and mechanical micromanipulations. Many preparation protocols are currently available starting with the gentle hydration method, originally described by Reeves & Dowben [17], using electric fields at low salt concentration as originally introduced by Angelova *et al*. [18], or at biologically relevant salt concentration [19], or via gentle spontaneous swelling [20], which is improved when performed on a polymer gel [21]. GUVs can be prepared with a single type of lipid, but also with synthetic lipid mixtures or natural lipid extracts. These systems have been particularly instrumental for the study of macroscopic phase separation in lipid mixtures [9,22,23] (figure 1*d*), showing essential differences between these systems at thermodynamic equilibrium and lipid clusters (rafts) in cells [24]. GUVs can even be formed by electroformation from native membrane extracts purified from cells [19]; however, how much of the initial membrane organization and of the protein activity can be preserved remains to be clarified.

Important steps towards more complex membranes were achieved when methods have been set up for *reconstituting biological machines* into GUVs, namely ion channels, ion pumps and transporters. These transmembrane proteins can be reconstituted in GUVs if the initial film consists of small proteoliposomes [25–30], by direct incorporation, or by fusion of small vesicles containing the proteins, using detergents [31] (figure 1*d*). The same type of method also applies to GUVs containing adhesion proteins such as integrins [32]. The published methods aim at being general, but practically, they have to be slightly adapted for each new protein reconstitution nevertheless.

Planar bilayers can be formed over solid substrates (*supported bilayer*) [33–35] with limited interactions in the presence of a polymer ‘cushion’ [36]. Because of their geometry and the vicinity of the substrate, they represent very convenient systems for atomic force microscopy, total internal reflection fluorescence and reflection interference contrast microscopy imaging as well as for surface plasmon resonance (Biacore). ‘Proteo-bilayers’ can be formed either from fusion of small proteoliposomes on the solid, or using direct incorporation [37]. In addition, supported bilayers can be prepared in microfluidic chambers, allowing to flow in proteins and biological molecules that bind to the bilayer; thus, they are routinely used as biosensor platforms. From the bioinspiration point of view, supported bilayers have been useful to understand, for instance, how spatial protein patterns are formed that lead to the proper localization of the proteins involved in cell division *in vivo* [38,39] (figure 1*c*). It is now possible to obtain *suspended bilayers* spanning over an array of holes of about 1 μm diameter formed in a silicon substrate [40]. In this geometry, if transporters are present in the membrane, the free volume on the cavity side prevents the accumulation of ions or molecules after crossing the membrane; electrical access is also possible to measure transmembrane potentials [41].

In cells, membranes are constantly remodelled and deformed in order to achieve various functions (e.g. endocytosis/exocytosis, trafficking, motility, cytokinesis, but also during infection by pathogens). *Membrane deformations* result from interactions with proteins, e.g. cytoskeletal filaments or nanomachines (molecular motors) that pull on membranes when moving along their cytoskeletal track [42]. Biomimetic systems and theoretical models coupling membrane mechanics and protein–membrane interactions have been crucial in the past decade to quantitatively explain how these deformations occur. It was possible to mimic the formation of dynamic membrane tubules by microtubule- and actin-related motors [43,44] by attaching purified motors to GUVs sedimented on immobilized microtubules (see [45] or [46] for reviews) or on actin filaments [47] (figure 1*b*). Liposome membranes can be functionalized with protein ligands, or with specific charged lipids (PS, phosphoinositides such as PIP2), allowing the recruitment and binding of proteins (figure 1*d*). Membrane-shaping proteins have been particularly investigated either by evaluating their affinity for curved membranes with the SLiC assay (see above), or by measuring their enrichment (or depletion) and their mechanical action on membrane nanotubes mechanically pulled from GUVs (see [10,48–50] for reviews (not exhaustive)). Membrane scission can also be studied using similar methods [51].

From a cellular building-up perspective and for a deeper understanding of the various consequences of *membrane–cytoskeleton interactions*, it is logical to try to reconstitute synthetic membrane systems with cytoskeletal filaments (figure 1*d*). So far, many attempts have been made to reconstitute an actin cortex. This is a hard task: initially, actin filaments were encapsulated in GUVs, but not anchored [52] to the membrane. Next, they were bound to the membrane, but on the external side [53]; in the presence of myosin 2, even actin contractility could be reproduced [54]. Only recently, a membrane-bound actin network (not contractile) was finally reconstituted into a GUV [55]. The actin-based motion of some pathogens such as *Listeria* was similarly reproduced by attaching actin nucleators to the surface of beads or vesicles (for a review, see [56]). Microtubules are much more rigid filaments; they have also been encapsulated into GUVs resulting in very deformed shapes that have been described as lemon-like or cherry-like [57]. Eventually, less conventional cytoskeletal systems have been reconstituted on GUVs such as FtsZ, a prokaryotic tubulin homologue [58].

Schwille's group has started to reconstitute bacterial *cytokinesis in vitro*. As mentioned above, with supported bilayers and modelling, Schwille and co-workers [38,39] could explain how protein gradients are established (figure 1*c*). They have gradually mimicked the cell confinement by using photolithography and patterns of membrane on the substrate [59]. Soft microfabrication was instrumental to obtain compartments with dimensions similar to bacteria and coated with lipid membranes, providing thus a sort of artificial cell where coordinated positioning of proteins involved in cell division was reproduced [41]. However, cell division was not observed in cell-free systems so far.

*High-throughput methods* have been applied for the preparation of GUVs in large number and with a systematic approach. Some are based on inverted emulsions that cross a lipid monolayer interface [60,61] and allow for protein encapsulation [55,62]. When this method is coupled to microfluidics, monodiperse GUVs can be obtained [63]. Even membranes of asymmetric composition can be prepared [64]. Alternatively, jetting of biological solutions through a bilayer spanning over a large hole can also produce a large number of GUVs of uniform sizes [65,66]. An output of the droplet technology arose from the possibility to form bilayers at the interface between doublets of aqueous droplets in oil containing lipids [67] or alternatively with vesicles present in the water droplets [68]. With this method called ‘droplet interface bilayer’, it has also been possible to directly incorporate proteins into the membrane when a cell-free system is encapsulated (see §2.2) [69]. However, as we discuss in the following, these methods although very attractive have some drawbacks.

### 2.2. Limitations of these systems for reaching biological complexity

*Biochemistry* is a strong limiting factor for the development of sophisticated bioinspired systems. Of course, proteins have to be purified and must remain correctly folded and active after fluorescent labelling (often used for protein detection); in the case of membrane proteins, they must additionally stay functional after reconstitution into membranes. This essential step requires robust biochemical protocols and gifted scientists!

The aforementioned high-throughput methods often involve the presence of oil during the preparation process: water droplets are emulsified or dispersed in an oil matrix, or the lipids are solubilized in a non-polar solvent for the preparation of the suspended bilayer used in the jetting method. Oil traces remain trapped in the bilayer when aqueous droplets cross the lipid-covered interface between the oil and water. This *bilayer contamination* was evidenced by mechanical experiments with the nanotube assay showing that the intra-bilayer friction was changed [70]. Black lipid membranes also tend to keep traces of the apolar solvent.

As we mentioned before, reconstitution of dynamical actin structures inside GUVs, growing from the membrane and mimicking reasonably well an actin cortex or filopodia is challenging. Adding contractility with myosins represents another layer of complexity. Many proteins are involved in actin dynamics and spatial organization. Their precise stoichiometry is important, and the controlled reconstitution of the different possible organizations, even in the absence of a membrane, is a full research field in itself (see for instance [71] for some appreciation of the richness of this problem). Nucleation of actin structures at a lipid bilayer interface adds even more complexity to the task. The number of proteins to include into the biomimetic assay increases, because proteins connecting the membrane and the actin filaments or promoting their polymerization from the membrane have to be bound to the membrane. Moreover, the experimental conditions are complicated by the fragility of the lipid bilayer that can rupture if osmotic balance is not respected or when tension is too high. Moreover, once all these components are incorporated inside a GUV, concentrations cannot be changed anymore. Thus, many experiments have to be performed to screen this complex phase diagram.

Another important problem must be addressed in the future: independent modules representing specific aspects of cell functions can be carefully engineered, but some incompatibilities subsist so far. For instance, it is possible to produce GUVs with transmembrane proteins reconstituted in the lipid bilayer on the one hand or some internal cortex on the other hand. However, the encapsulation methods generally imply an intermediate stage with a lipid monolayer around the droplet, incompatible with transmembrane protein preservation. Conversely, if the cytoskeleton proteins are encapsulated in GUVs first, post-reconstitution of membrane proteins is possible in principle [31], but it involves detergents and possibly leaks of some of the GUV's components (the smallest) primarily trapped in the GUV lumen. A possible solution would be to prepare bilayer lipid membranes by bursting giant proteoliposomes onto a hole and use the jetting method, with the new issue that this would be a ‘one-shot’ experiment, because the bilayer cannot be reformed easily, thus methods for parallelization would have to be developed.

Like cytoskeleton assemblies that are mainly studied independently of membranes, the nucleus is also a distinct world with its own variety and complexity. In the age of molecular biology, research had been focused on chromatin and DNA using biochemical approaches. Although considerable effort has been put into the investigation of, for example, spatial plasticity of the genome within the nucleus and mechanical measurements to characterize the mechanical properties originating from a very peculiar network of fibrillary proteins in the past two decades [72], the role of the nuclear envelope which serves as a determining feature of the nucleus still poses a lot of open questions. How lipid heterogeneity is maintained in the light of constant exchange with the endoplasmic reticulum, how it might affect nuclear organization and control the activity and localization of nuclear proteins, e.g. lamins and chromatin, is unresolved [73,74]. Furthermore, its fragmentation during cell division and the implications thereof for genome maintenance and repartition remain unclear [75]. Technologies that have advanced the field of membrane research in cell biology will be useful to advance this research and foster a collaborative environment between disciplines, such as we have observed between cell biology and biophysics.

## 3. Model membrane-based systems for synthetic biology and medical applications: possible synergies

### 3.1. Some common applications of model membrane systems

Model membrane systems as those described in §2, but in their simplest version, are used more or less routinely for medical applications and also in synthetic biology.

#### 3.1.1. Encapsulation and drug delivery

**Figure 2.**
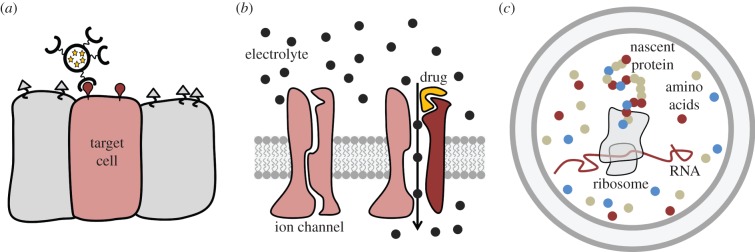
Application of model-membrane systems. (*a*) Functionalized liposomes filled with pharmaceutical compounds can be used for targeted drug delivery. (*b*) Lipid bilayer systems with reconstituted transmembrane proteins, e.g. ion channels, offer high throughput in the screening of compounds that are modulating the gating of these channels. (*c*) Confining *in vitro* protein synthesis in the small volume of GUVs allows for straightforward production of highly concentrated products without the need for prolonged purification.

Liposomes are commonly used as drug transporters with encapsulated pharmaceutical products (figure 2*a*). Considerable efforts have been made on the grafting of hydrophilic polymers (polyethylene glycol) on their membrane to extend their circulation lifetime in the body and prevent their rapid elimination by the immune system. Many ‘stealth’ liposome formulations are on the market [76]. Their efficiency can be increased by adding specific ligands for targeting the liposome delivery to a specific organ that has to be treated [77]. Lipids can be replaced by copolymers and liposomes can be controlled to release their content only upon specific stimuli, e.g. external ones such as light or internal ones such as pH [78].

#### 3.1.2. Supported bilayers and biosensors

Supported bilayers containing membrane proteins are convenient platforms for detecting substances in biological liquids when they are coupled to a detection method, e.g. electrical [79], electrochemical [80] or optical (surface plasmon resonance) [81] (figure 2*b*). Miniaturization and integration of arrays of sensors make membrane chips very suitable for drug screening [82]. Practically, they can be made more resistant by tethering the bilayer to the solid support [83]. Similar to liposomes, lipids can be substituted by copolymers to achieve a longer stability, but, nevertheless, with reconstituted proteins such as channels [84]. Moreover, because so many efforts are devoted to the improvement of *in vitro* reconstitution of biological systems, we could expect that these progresses will be beneficial to the biosensor field.

#### 3.1.3. Lipid encapsulated protocells

An interesting progress was made in synthetic biology when a cell-free expression system was encapsulated inside a GUV and that fluorescent protein expression in the GUV lumen could be followed optically (figure 2*c*); moreover, to keep access to an ATP reservoir for sustained activity, GUVs were incubated in an ATP-containing buffer and pore-forming toxins were reconstituted into the membrane [85]. Interestingly, owing to the small internal volume of GUVs, substantial concentration of proteins can be obtained in a short period of time when compared with the same expression method in bulk where products become diluted. This constituted an important step, but, nonetheless, the vesicles were used as passive containers in these assays. As we will see in the following, more advanced systems are now in development.

### 3.2. Synergy between biomimetic system engineering and synthetic biology

Synthetic biology develops increasingly creative methods, often bacteria-based, to produce for instance new fuel sources [3], drug precursors for low-cost and diversified pharmaceutical products, or new chemical products that could otherwise be obtained only after tedious chemical synthesis. As is often the case with basic sciences, we could expect that the knowledge and technology developed while engineering new cell-free systems mimicking cell ‘modules’ can also contribute to the further improvement of systems for synthetic biology and in medical applications. Moreover, even if the cell modules currently produced still seem basic, it might be difficult today to imagine their future technological developments. At this stage, it still remains a dream to fully reconstruct a functioning cell de novo and the ‘artificial cell’ project is still in its infancy. We recommend reviews from Noireaux *et al.* [62] and from Schwille [5] for insights into what should be achieved to set up rudimentary synthetic organisms inspired by biology. In the engineering perspective to eventually construct a cell, we must first build up individual functional modules specialized in a single essential function, e.g. production of the cell's building blocks, generation of a sustainable energy system, cell division or cell motion.

#### 3.2.1. Building a self-sustained factory

**Figure 3.**
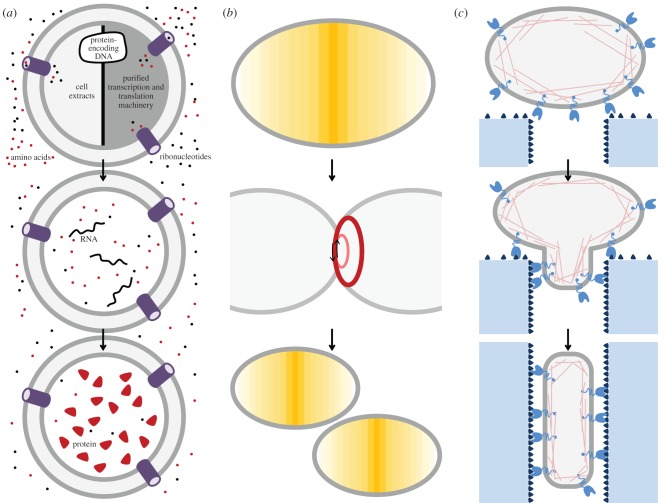
Towards functional cell modules. (*a*) Encapsulation of either purified components or cell extracts into small containers that are permeable for the required building blocks and energy carriers allows the sustained activity of bioreactors in which high yields of protein can be achieved providing the protein encoding DNA. This requires the functional integrity of both a transcriptional and translational system. (*b*) Autonomously dividing cell module. Two-dimensional confinement results in the generation of a protein pattern that leads to the definition of a ‘centre’. Recruitment of proteins towards this centre leads to formation of a contractile ring that creates a furrow, which finally leads to fission after recruitment of curvature sensitive proteins. (*c*) Autonomously crawling cell module. The cell module consists of adhesion molecules that are present on the outside and a dynamic actin network on the inside. After initial adhesion to a substrate, symmetry breaking is caused by geometrical constraints and an adhesion gradient (higher concentration of adhesion molecules or stronger interacting adhesion molecules inside channel). The actin dynamics are now able to generate a force towards the channel, which results in net movement of the cell module until completely having entered the channel.

The long-term goal is to build a sort of ‘factory-GUV’ that would (i) be able to produce its own energy (ii) to generate molecules or new proteins from amino acids that could be present on the GUV exterior and supplied to the GUV interior by transporters present into the lipid bilayer (iii) whereas other carriers would take care of the wastes (figure 3*a*). A system able to express its own ATP-synthases and carriers and to directly incorporate them into the GUV membrane would be even better. Along these directions, transcription and translation machineries must be isolated and encapsulated into GUVs. The first method consisted of using cell extracts where the endogenous genetic material was replaced by bacteriophage RNA polymerase. Next, the protein synthesis using recombinant elements system (PURE system), a minimal synthesis system that uses only a set of purified components [86], has been really instrumental and has allowed the incorporation of membrane proteins in different model membrane systems, including liposomes (see [87] or [88] for recent reviews). In addition, in a first generation, energy supply and nutrient molecules can be located outside the vesicle container and transported through pores or transporters across the bilayer [87] after proper adjustment of the lipid composition [89]. However, with progress in membrane protein reconstitution (§2.1), it might become possible to reconstitute the machinery for ATP production. In addition to the ATP-synthase incorporation, a pH gradient has to be prepared which might be possible if a multi-compartment vesicle is formed. So far, separate compartments have been prepared using the phase transfer of multiple droplets, that can communicate using toxin pores [90], but methods have to be adapted to GUVs.

#### 3.2.2. Dividing vesicles

In order to generate a self-dividing vesicle, three molecular machineries are essential (figure 3*b*). First, the place where fission will occur has to be defined intrinsically, something that could be achieved by self-establishing protein gradients as has been demonstrated in the group of Schwille for the Min system from bacteria (see §2.1). Next, the determination of a division site needs to engage the recruitment of accessory proteins that form a contractile ring structure linked to the membrane, which will lead to furrow formation at the pre-established division site [91,92]. In a last step, the recruitment of proteins that drive the final scission at the furrow into two independent daughter cells can be driven ideally by curvature sensitive binding at a defined furrow ingression, thus making it independent of more complex biochemical cascades, e.g. the ESCRT machinery [93]. At this stage, two individual ‘daughter modules’ can undergo the same process again with effective protein concentrations as a limiting factor. Another issue to face is the rapid reduction of size of the ‘pseudo-cells’ if no systems for lipid production and GUV size regulation are included. This is precisely the type of challenge faced when building up protocells for understanding the origin of life [94].

#### 3.2.3. Self-propelled vesicles

A minimal module for self-propelled vesicles requires the presence of two cellular machineries and a geometrical constraint. We propose one possible solution. Initial adhesion of the module is driven by adhesion of transmembrane receptors to their ligands that are presented on a fixed substrate. At this stage, a dynamic actin cortex beneath the cell membrane does not generate any effective propulsion force, as the module is still isotropic. By driving the adherent module to face a channel (figure 3*c*), either by varying the concentration of available ligands, or varying the affinity of ligands between the channel inside and its outside limits, symmetry breaking occurs and the strong adhesion at the channel walls provides enough friction for the actin dynamics to polarize the module towards the channel. In a ratchet-like behaviour, the module will thus be able, driven by adhesion and protrusive forces caused by actin, to fully enter the channel, at which point symmetry is again re-established and movement should stall. This module would thus recapitulate some of the hallmarks of haptokinetic movement as observed in two-dimensional migration.

## 4. Perspectives and questions

So far, we have considered how an artificial cell could be built by assembling together purified biological elements or by producing them *in situ*. We have also discussed how these approaches can be useful to understand cell functioning, how synthetic biology and cell biophysics can mutually benefit from their own advances, and potentially how cells were originally built during evolution. This manufacturer-type approach might be a very long-term adventure with many dead ends. On the way, it might be necessary to consider alternatives to building up from scratch each element and use some already built natural modules, in particular membranes.

A traditional but yet efficient way to bypass purification and reconstitution of proteins in GUVs is to *express exogenous proteins in frog* oocytes (*Xenopus laevis*). This method has been widely used for the expression and characterization of different types of proteins, including ion channels and membrane receptors [95]. This method takes advantage of the ability to efficiently translate exogenous mRNA into proteins, of the large size of the oocytes (1–2 mm) that are thus suitable for microinjection, and of the possibility to inject multiple species of mRNA. It is particularly convenient when studying the biophysical properties of proteins expressed in the membrane of the oocytes. Some chemical treatments allow removing the cells enveloping the oocytes giving access to the membrane, which can then be manipulated for a good period of time. Instead of doing *in situ* experiments on oocytes, it would be interesting to take patches out of them which would become the envelopes of the future protocells. This implies that methods have to be designed to convert membrane patches into giant liposomes. Nevertheless, the large surface of oocytes could then represent a wonderful source of functional membranes.

A very promising method to obtain membranes with lipid and protein compositions close to those of cells is the direct formation of GUVs by *cell blebbing* owing to the detachment of the plasma membrane from the cortical cytoskeleton. Thus, GUVs have the exact composition and asymmetry in terms of lipids and membrane proteins as the native plasma membrane, but no interaction with actin filaments (for a review, see [96]). These blebs can be obtained (i) by chemical treatment forming giant plasma membrane vesicles [97], with the problem that membrane proteins can be cross-linked or (ii) by cell swelling forming plasma membrane spheres, which preserves protein distribution in the membrane [98]. This method is promising, but has still to be developed further to extend the range of cells from which the blebs can be collected.

In the future, these alternatives to purely synthetic biology should be seriously considered in parallel. Similar approaches were used in the past when cytosolic extracts were used before essential proteins were identified and isolated. Although the membrane components will not be as well controlled as in reconstituted systems, it could help building hybrid systems containing natural and synthetic elements, like a cyborg cell. In this direction, more progress in cell organelle purification is also necessary, because it could be interesting to include for instance already-made mitochondria, or a nucleus into giant liposomes.

We hope that this review provided some insights, although far from being complete, about the use of reconstituted systems to address cell biological problems as well as the engineering of a cell. We propose to open the discussion with some questions:

— What can we still do with synthetic systems?
— Where do we stop building up?
— Which are the alternative solutions?
